# Unveiling the Future of Meat Packaging: Functional Biodegradable Packaging Preserving Meat Quality and Safety

**DOI:** 10.3390/polym16091232

**Published:** 2024-04-28

**Authors:** Phanwipa Wongphan, Khwanchat Promhuad, Atcharawan Srisa, Yeyen Laorenza, Chayut Oushapjalaunchai, Nathdanai Harnkarnsujarit

**Affiliations:** 1 Department of Packaging and Materials Technology, Faculty of Agro-Industry, Kasetsart University, 50 Ngam Wong Wan Rd., Latyao, Chatuchak, Bangkok 10900, Thailand; phanwipa.w@ku.th (P.W.); khwanchat.pro@ku.th (K.P.); atcharawan.sri@ku.th (A.S.); yeyen.la@ku.th (Y.L.); chayut.ou@ku.th (C.O.); 2 Center for Advanced Studies for Agriculture and Food, Kasetsart University, 50 Ngam Wong Wan Rd., Latyao, Chatuchak, Bangkok 10900, Thailand

**Keywords:** meat products, biodegradable, functional packaging, food preservative, meat quality, flavor contamination, odor

## Abstract

Meat quality and shelf life are important parameters affecting consumer perception and safety. Several factors contribute to the deterioration and spoilage of meat products, including microbial growth, chemical reactions in the food’s constituents, protein denaturation, lipid oxidation, and discoloration. This study reviewed the development of functional packaging biomaterials that interact with food and the environment to improve food’s sensory properties and consumer safety. Bioactive packaging incorporates additive compounds such as essential oils, natural extracts, and chemical substances to produce composite polymers and polymer blends. The findings showed that the incorporation of additive compounds enhanced the packaging’s functionality and improved the compatibility of the polymer–polymer matrices and that between the polymers and active compounds. Food preservatives are alternative substances for food packaging that prevent food spoilage and preserve quality. The safety of food contact materials, especially the flavor/odor contamination from the packaging to the food and the mass transfer from the food to the packaging, was also assessed. Flavor is a key factor in consumer purchasing decisions and also determines the quality and safety of meat products. Novel functional packaging can be used to preserve the quality and safety of packaged meat products.

## 1. Introduction

Petroleum-based plastic packaging is environmentally unfriendly because petroleum plastics produce high carbon emissions during their extraction and refinement processes [1]. Petroleum-based plastic packaging is poorly biodegradable and can take more than a hundred years to degrade. Environmentally friendly bioplastic packaging is now widely used in the food industry [2,3], and the biocircular green (BCG) economic model has promoted increasing consumer demand for biodegradable plastics [1,2,3]. In recent years, the packaging industry has undergone a transformative shift towards sustainability and eco-conscious solutions. With growing concerns about environmental degradation and plastic pollution, there is a pressing need for innovative packaging technologies that preserve the quality and safety of food products and minimize their environmental footprint [3,4,5,6]. In this context, the emergence of functional biodegradable packaging represents a groundbreaking advancement.

Bioplastic materials represent a significant stride toward sustainability in packaging and material production. These materials are derived from renewable biomass sources such as plant starches, cellulose, or even agricultural waste, offering an eco-friendly alternative to traditional plastics and also reducing carbon emissions. Bioplastics can be categorized based on their composition, which includes biobased, biodegradable, or both [3,5,7,8]. Bioplastic materials present a viable alternative to traditional petroleum-based plastics. Bioplastics represent a burgeoning field within the broader realm of sustainable materials science. The state of the art in bioplastic materials is characterized by ongoing advancements in feedstock utilization, biodegradability, performance properties, and processing technologies [9,10]. These developments have contributed to the growing adoption of bioplastics across various industries, fostering a more sustainable approach to plastic production and consumption. Functional bio-material packaging offers a holistic approach to addressing the dual challenges of food preservation and environmental sustainability. When engineered with functional properties tailored to meat products, such as barrier enhancement, antimicrobial efficacy, and active packaging features, biodegradable packaging can revolutionize the way meat is packaged, distributed, and consumed [8,11,12,13].

The critical factors at different stages of the meat supply chain that effect the shelf life of packaged fresh meat and potential strategies to improve its quality have been reviewed [14]. Furthermore, the utilization of active functional packaging materials in the meat industry has garnered significant attention due to their potential to enhance food safety, extend shelf life, and maintain product quality. The potential of applying essential oils, nanoparticles, and natural antioxidants in functional meat packaging has been reviewed [15,16,17]. However, a comprehensive review of the meat quality standards and packaging necessities needs to be thoroughly evaluated. This review evaluates the application of alternative active packaging materials, particularly in the realm of meat packaging, including chemical additives and natural extracts. We also focus on evaluating the utilization of biodegradable packaging materials such as PBAT, PLA, and starch, whether used singly or in blends. Moreover, this review investigates the factors contributing to meat spoilage and flavor/odor contamination of meat products by packaging components, offering insights into the strategies for improving packaging performance and ensuring consumer safety.

## 2. Major Challenges for Meat Products

Meat products are an important source of protein and rich in key nutrients, minerals, vitamins, and water, thereby supporting human health. Fresh meat is a highly perishable food due to the composition of meat, while its high water activity (aw > 0.95) promotes the propagation of spoilage and pathogenic microorganisms. The quality and shelf life of meat products are determined by their color, odor, texture, and flavor. The factors impacting meat’s deterioration include holding time, temperature, storage conditions, moisture, pH, relative humidity, water activity, and atmosphere. Both intrinsic and extrinsic factors play critical roles in meat quality changes [18].

### 2.1. Meat Composition

Meat is composed of macromolecules including protein, fat, minerals, and vitamins. Its protein content ranges from 16 to 22% and averages 18.5% of the weight of muscle. Meat is very low in carbohydrates at 0.5–1.5%, with its lipid and fat contents ranging from 1 to 13% depending on the muscle type and animal age. Meat also contains non-protein nitrogenous substances (phosphate, peptides) and other non-protein substances (minerals, vitamins) at 1.7% and 0.85%, respectively. The percentage of water (approximately 75%) in the muscle plays a key role in supporting microorganism growth [19,20].

### 2.2. Quality and Safety of Meat Products

Meat quality is described according to its appearance, texture, flavor, odor, and color, with its nutritional value resulting from chemical/biochemical, microbiological, and physical reactions. Interactions between both intrinsic and extrinsic factors impact the occurrence of reactions that affect the quality, shelf life, and safety of food products [21].

#### 2.2.1. Discoloration

Meat color is an important factor in purchasing decisions because consumers use color to discern the freshness of meat. Color preservation of meat in the food industry can be achieved by introducing additives such as salt and nitrite and using modified atmosphere packaging (MAP) and vacuum packaging [20,22]. Myoglobin is a complex muscle protein and gives meat its red color. The color of myoglobin is determined by oxidation–reduction (redox) reactions, with a reduced ferrous form (Fe^2+^) or an oxidized ferric form (Fe^3+^). Myoglobin can exist in four redox states, namely (i) oxymyoglobin (Fe^2+^), (ii) metmyoglobin (Fe^3+^), (iii) deoxymyoglobin (Fe^2+^), and (iv) carboxymyoglobin (Fe^2+^), which cause the meat color to be bright red, brown, purple, or cherry red, respectively [21,23].

**Figure 1:**
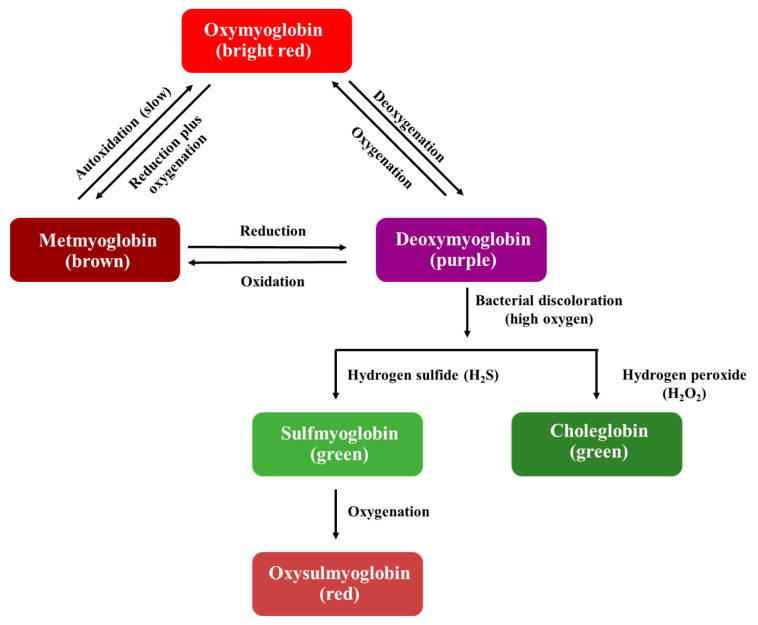
The interrelationships between meat pigments according to oxidation–reduction myoglobin reactions. Modified from Kerry [20], Robertson [21], and Mancini and Hunt [23].

The interrelationships of myoglobin as primarily responsible for meat color are shown in Figure 1. The reversible reaction between oxymyoglobin and deoxymyoglobin is explained by oxygenation and deoxygenation, attributed to the amount of oxygen present. Deoxymyoglobin is detectable as purple on meat’s surface, typically associated with vacuum-packaged products because of their rapid oxygen change to a low oxygen concentration (0.5–1.0%), while oxygenation occurs when myoglobin is rapidly exposed to oxygen, generating a red color.

Oxidation occurs when heme iron is converted from the ferrous into ferric form. Metmyoglobin is a stable form that can exist either as oxymyoglobin or deoxymyoglobin. The formation of metmyoglobin from oxymyoglobin results from the lipid oxidation rate, which depends on oxygen partial pressure, temperature, pH, meat activity, and microbial growth. The reduction reaction works in the opposite way to oxidation. All the reactions in this cycle are reversible, with different discoloration according to the storage conditions, which depend on intrinsic factors, including pH, type of muscle, and meat activity, and extrinsic factors, including temperature, oxygen content, and the environment [20,21,22,23]. Deoxymyoglobin undergoes a wide variety of reactions, leading to discoloration in meat. Bacterial discoloration occurs in the log phase of microbial growth, when a high oxygen content affects the growth of aerobic bacteria and causes the formation of myoglobin. Some bacteria produce hydrogen sulfide (H_2_S) and hydrogen peroxide (H_2_O_2_), which react with deoxymyoglobin to produce sulfmyoglobin and choleglobin, respectively. The resulting pigment gives meat a green color. Oxysulfmyoglobin is generated when meat is exposed to high oxygen levels (oxygenation) and causes a red appearance [21,24]. The packaging types used to preserve a red color in meat are shown in Table 1.

Color stability in meat products is mostly achieved using modified atmosphere packaging to alter the concentrations of nitrogen, oxygen, carbon dioxide, and carbon monoxide. Meat is also immersed in various solutions such as tea catechins, Vitamin E, thymol, carvacrol, and grapefruit seed extract to reduce oxidation and preserve color stabilization. However, scant research has addressed the use of additives to develop functional packaging and improve the color stability of meat and poultry products. Copious research is now focused on novel functional packaging for extending the shelf life and preserving the quality of meat products.

#### 2.2.2. Texture

Texture is one important indicator of the quality of meat products influencing consumer acceptability and satisfaction. Texture describes meat’s firmness, tenderness, and juiciness depending on its physicochemical and biochemical changes [20]. Firmness and tenderness are complex quality parameters, described as the mechanical strength of muscle and connective tissue, which is affected by both antemortem and postmortem factors. Intrinsic antemortem factors affecting meat tenderness include the animal species, connective tissue, muscle fiber cells, and fat, while postmortem factors impacting meat’s texture include the temperature and pH of the muscle, proteolysis, and its water-holding capacity [19,20,30]. Protein oxidation is associated with both physical and chemical changes in meat quality and is linked to meat tenderness. Protein oxidation occurs through a loss of enzyme activity and the formation of amino acids, leading to a loss of amino acid structure, a reduced water-holding capacity, and decreased protein solubility [20].

Texture can be improved using salt marination, high-pressure processing, irradiation, and a modified atmosphere to reduce water loss and increase products’ shelf life [31]. Seyfert et al. [32] investigated the impact of modified atmosphere packaging on the oxidative and sensory properties of beef. The results showed that a low oxygen content decreased oxidation and improved its tenderness, while Kim et al. [33] found that modified atmosphere packaging (70% O_2_/30% CO_2_) reduced the cross-linking of myosin chains through disulfide bonding and the content of protein thiols, indicating protein oxidation, with reduced tenderness and juiciness of the meat. Protein oxidation or deformation modifies the texture of meat.

#### 2.2.3. Flavor and Odor

Flavor and odor are factors used to determine the quality of meat and impact consumer perception. Flavor and odor are important quality attributes of muscle foods and comprise the two sensations of taste and aroma or smell. The characteristics of flavor and odor depend on the animal species, temperature, and method of cooking. Beef, chicken, and pork contain many organic compounds, such as hydrocarbons, alcohols, aldehydes, ketones, carboxylic acids, esters, lactones, ethers, furans, pyridines, pyrazines, pyrroles, oxazoles and oxazolines, thiazoles and thiazolines, thiophenes, and other sulfur- and halogen-containing substances [34]. In fresh meat, flavor and odor denaturation occur as an off flavor/odor and rancidity during storage as a result of lipid oxidation [20,34,35].

Lipid oxidation causes quality deterioration in flavor and odor, as well as in color and texture, as described previously. Lipid oxidation occurs due to fatty acid and phospholipid exposure to oxygen and is accelerated by light and catalysts such as free iron. Free iron is an important catalyst because it is abundant in meat muscle. Lipid oxidation consists of three steps: (i) initiation, (ii) propagation, and (iii) termination [36,37].

(i) Initiation step: Free radicals such as the hydroxy radical (•OH), abstracted from an unsaturated fatty acid, are accelerated by free iron to form the lipid peroxyl radical, which then undergoes molecular rearrangement to form conjugated dienes or trienes.

(ii) Propagation step: The lipid peroxyl radicals from the initiation step react with molecular oxygen to form peroxyl radicals (•OO), which then abstract hydrogen from adjacent lipid molecules, resulting in lipid hydroperoxide (OOH). This then reacts with molecular oxygen to form new peroxyl radicals, and the reaction continues.

(iii) Termination step: Peroxyl radicals (•OO) react with radicals or other non-radical compounds (antioxidants) to form a non-radical product.

The use of natural antioxidants to increase meat’s oxidative stability is a topic of great interest, whereby an antioxidant compound transfers a hydrogen atom to the radical derived from lipid oxidation. This reaction neutralizes the lipid radical and creates a new radical from the antioxidant compound [36].

#### 2.2.4. Microorganisms

Microorganisms play an important role in meat quality and safety because microbial contamination or microbial growth is of the highest concern for consumers. Meat and poultry have a high water activity (aw > 0.95) and are highly perishable products. Microbes including bacteria, yeast, and mold lead to spoilage and pathogens. Microorganisms break down the fats, carbohydrates, and proteins in the meat muscle, causing oxidation and chemical deformation, resulting in off flavors, off odors, slime formation, texture change, gas production, and discoloration, which impact consumer acceptability [38,39].

The microorganisms mostly found in meat include *Arthrobacter* spp., *Acinetobacter* spp., *Aeromonas* spp., *Staphylococcus* spp., *Enterococcus* spp., *Moraxella* spp., *Psychrobacter* spp., *Pseudomonas* spp., *Cladosporium* spp., *Geotrichum* spp., *Mucor* spp., *Rhizopus* spp., *Sporotrichum* spp., *Thamnidium* spp., *Candida* spp., and *Torulopsis* spp. [38,39,40]. Indications of the microbial population are expressed as organisms per square meter or as organisms per gram. Generally, the initial microbial counts in meat range from 10^2^ to 10^5^ CFU/cm^2^, and meat starts to spoil at a total viable count of 10^6^ CFU/cm^2^, resulting in the production of an off flavor, while at 10^8^ CFU/cm^2^, the meat shows slime on its surface and discoloration [41,42].

## 3. Bioplastic Materials

**Figure 2:**
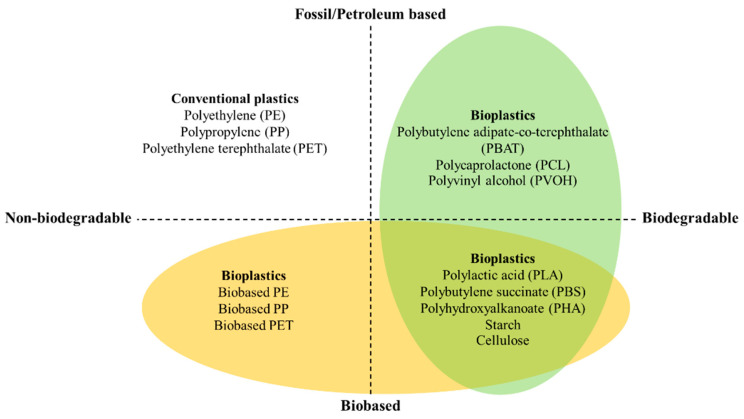
Type of plastic classified by resource (fossil-based or biobased) and biodegradable characteristics. Modified from *Bioplastics* (2021) [44].

Bioplastics are sustainable materials produced from renewable resources or obtained from biomass that are used to produce environmentally friendly packaging with reduced carbon emissions. Bioplastic, biobased, or biodegradable materials can be classified according to the type of plastic, as shown in Figure 2. The bioplastic materials that are fossil-based or made according to chemical synthesis through polymerization include polybutylene adipate-co-terephthalate (PBAT), polycaprolactone (PCL), and polyvinyl alcohol (PVOH). Bioplastics are also made from natural resources such as sugar cane, starch, cellulose, and seaweed. Cellulose-based material can be prepared using 2,2,6,6-tetramethylpiperidine-1-oxyl (TEMPO)-oxidized bacterial cellulose powder [43]. The bioplastics produced from microorganism fermentation include polylactic acid (PLA) and polyhydroxyalkanoate (PHA). However, not all bioplastics made from biobased natural resources are biodegradable. Biodegradable materials undergo polymer transformation by biological organisms and can be easily cleaved through hydrolysis or enzymatic activity [5,6,13].

Bioplastic materials have diverse properties. This study focused on PBAT, starch, PLA, cellulose, and PBS as the biodegradable materials mostly used in the plastic industry and produced as flexible and rigid materials to support market demand [4]. The current market studies forecast the global production capacity of biodegradable materials of 1.14 million tons to reach 4.61 million tons between 2023 and 2028. This growth is stimulated by demand from Asia and the United States [4].

### 3.1. Polybutylene Adipate-Co-Terephthalate (PBAT)

**Figure 3:**
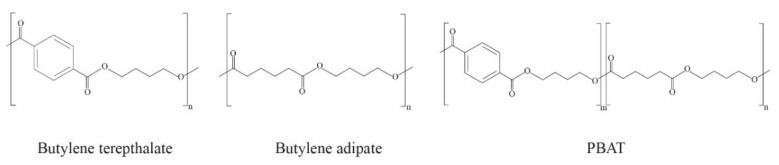
Structures of polybutylene terephthalate (PBT), polybutylene adipate (PBA), and polybutylene adipate-co-terephthalate (PBAT). (Reproduced with permission from Harnkarnsujarit et al. (2021) [5]).

PBAT is a random co-polyester produced according to the polycondensation reaction of adipic acid, 1,4-butanediol, and dimethyl terephthalate. PBAT is biodegradable and made from a synthetic petroleum base including two segments of (i) polybutylene adipate (PBA) and (ii) polybutylene terephthalate (PBT), containing both aliphatic and aromatic units, as shown in Figure 3. The fractions of the aliphatic and aromatic units affect the biodegradable rate and properties [45,46]. According to data from European Bioplastics [4], in 2023, the global production capacities of PBAT represented 4.6% of the overall bioplastic production, at more than 100 thousand tons [4]. PBAT has excellent mechanical properties compared to polyesters such as polylactic acid and polybutylene succinate, while the mechanical properties of PBAT show a high flexibility, similar to low-density polyethylene (LDPE). PBAT-based products have been widely used in many applications, such as shopping bags, garbage bags, and mulch films. However, the limitations of PBAT include high production costs and low transparency [3,46]. The improvement and development of PBAT properties will be discussed later.

### 3.2. Starch

**Figure 4:**
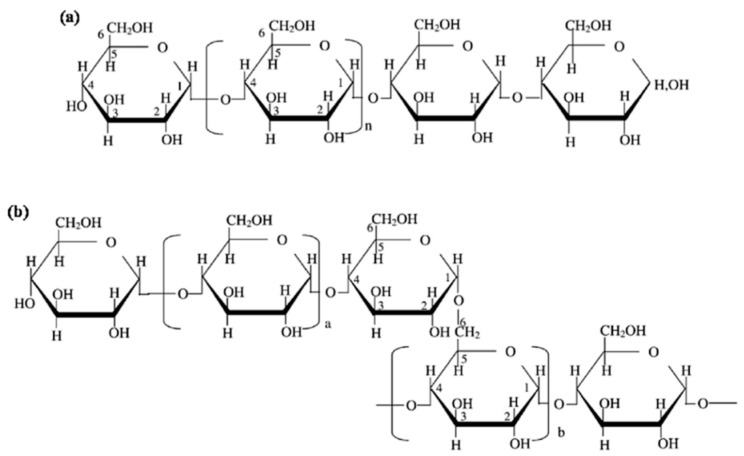
Structure of (**a**) amylose and (**b**) amylopectin. (Reproduced with permission from Amagliani, O’Regan, Kelly, and O’Mahony (2016) [50]).

Starch is a source of carbohydrates found in plants as energy reserve materials. This polymeric mixture consists of two main polymers, amylose (liner polymer) (Figure 4a) and amylopectin (branch structure) (Figure 4b). The structure of starch comprises D-glucopyranose units joined together by glycosidic bonds between α-1,4-glycosidic linkages and α-1,6-glycosidic linkages, as shown in Figure 4. The starch granules in semi-crystalline starch consist of crystalline and amorphous regions, which are derived from amylose and amylopectin, respectively. The amylose and amylopectin proportions depend on the source type, molecular size, chain length distribution, and degree of polymerization [47,48,49]. The proportions of amylose and amylopectin influence the fundamental and physicochemical properties of starch, with variations in composition and structures related to the diverse genotypic sources of starch.

Starch is used in the food industry as a thickener and stabilizer and also for the preparation of plastic materials because it is an abundant, low-cost, and biodegradable renewable resource. Starch films can be prepared from plasticized starch as thermoplastic starch (TPS) using plasticizers such as water, glycerol, and sorbitol to reduce their melting point because the melting temperature of starch granules is higher than the decomposition temperature (230 °C). Starch transforms from granules into TPS when heated at high temperatures, with high shear forces and added plasticizers contributing to the de-structure of the starch granules via extrusion processing [5,51,52].

A plasticizer is incorporated into the material to increase the flexibility and processability of starch and also reduce the glass transition temperature [53]. Molecules of the plasticizer penetrate the starch granules (amylose and amylopectin) to break the inner hydrogen bonding under high-temperature, high-pressure, and shearing conditions. Substitution through the starch–plasticizer interaction allows the starch network to be easily deformed and eliminates the starch–starch reaction because the plasticizer molecules are smaller and have higher molecular mobility than the starch molecules [54]. The most common plasticizers used in the processing of TPS are water and glycerol. The type and amount of plasticizer influence the films’ mechanical and barrier properties, thermal stability, and transparency.

### 3.3. Polybutylene Succinate (PBS)

**Figure 5:**
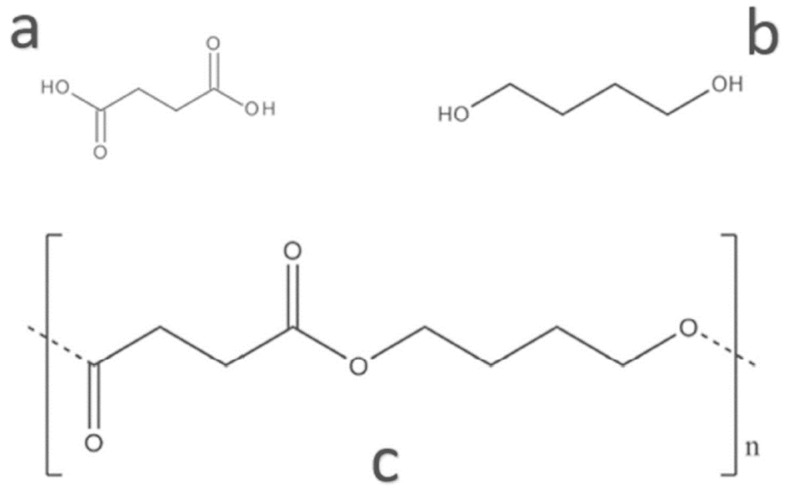
Chemical structure of (**a**) succinic acid, (**b**) 1,4-butanediol, and (**c**) polybutylene succinate (PBS) (Reproduced with permission from Aliotta et al. (2022) [55]).

PBS is an aliphatic co-polyester produced through the polycondensation of succinic acid and 1,4-butanediol, as shown in Figure 5. Monomers of PBS can be produced from fossil-based or renewable resources according to bacterial fermentation. PBS is a semi-crystalline polymer that influences the stiffness or mechanical strength, transparency, and flexibility of materials. The properties of PBS depend on its degree of crystallinity. PBS has similar properties and processability to polyolefins such as polyethylene (PE) and polypropylene (PP) with a low glass transition temperature and high elongation at break (more than 500%). PBS is widely used to produce packaging films, agriculture mulch films, and compost bags [13,55,56,57].

### 3.4. Polylactic Acid (PLA)

**Figure 6:**
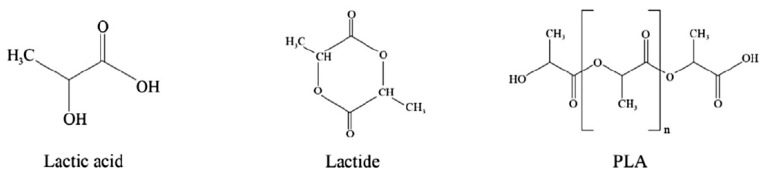
Chemical structure of lactic acid, lactide, and polylactic acid (PLA). (Reproduced with permission from Harnkarnsujarit et al. (2021) [5]).

Figure 6 shows the chemical structure of PLA. PLA is an aliphatic polyester that is synthesized through different polymerization processes, including (i) the polycondensation of lactic acid and (ii) ring-opening polymerization of lactide [58]. Direct condensation of lactic acid is easier in its synthesis and commercialization, but this process produces low-molecular-weight products. Ring-opening polymerization is mostly used for PLA synthesis to produce high-molecular-weight products [59]. Ring-opening polymerization of lactide involves enantiomers such as L-lactide and D-lactide, to produce poly(L-lactide) (PLLA) and poly(D-lactide) (PDLA), respectively. Both PLA forms have semi-crystalline structures with glass transition and melting temperatures at around 55 and 175 °C, respectively. PLA is highly brittle at room temperature and has poor thermal stability; therefore, it needs to be modified and blended with other polymers to improve these limitations [5,13].

### 3.5. Bioplastic Blends

Blending is an important process for modifying and improving the properties of bioplastic polymers. Pure biopolymers have limitations during processing and poor properties compared to fossil-based or conventional polymers [3]. The properties of bioplastic packaging, including PBAT, TPS, PBS, and PLA, are shown in Table 2.

Bioplastic limitations have led to the blending of various bioplastics to improve their properties and increase economic competitiveness. Different blending ratios influence the physical, chemical, morphological, and thermal properties of films. Zhai et al. [60] demonstrated the effect of the TPS/PBAT ratios on films’ chemical and physical properties. The compatibility of starch and PBAT improved by increasing the PBAT content from 10% to 50%, while the film strength and flexibility improved through blending with PBAT, which modified the barrier properties by improving the hydrophobic surface of the films. Zhang et al. [61] prepared bioplastic blends of PLA and PBS that improved the properties of pure PLA or PBS. They found that adding PBS increased the elongation at break and decreased the tensile strength by enhancing the distinctive PBS features of flexibility, while PLA acted as a rigid filler, with stiffness improvement. Garalde et al. [62] investigated the impact of TPS/PBAT film ratios of 20/80, 40/60, and 60/40 on the films’ morphological, mechanical, and thermal properties. The results showed that increasing the TPS/PBAT ratio to 40/60 led to an improved polymeric component distribution and an increased PBAT crystallization temperature, while the tensile properties of the TPS/PBAT films were reduced by increasing the proportion of TPS. Bumbudsanpharoke, Wongphan, Promhuad, Leelaphiwat, and Harnkarnsujarit [2] studied different PBAT/PBS ratios of 20/80, 40/60, 60/40, and 80/20 on microstructural modification, the degree of crystallinity, and relaxation temperatures. The bioplastic blended polymer had smooth and compact microstructures, causing compatibility and adhesion at the polymer interface. The barrier properties of the bioplastic blended film increased with an increasing PBS content because the degree of crystallinity followed a more tortuous path, with reduced permeation.

**Table 2:** Properties and applications of bioplastic packaging.

Bioplastic	Properties	Advantages	Disadvantages	Packaging Applications	References
PBAT	T_m_~110–125 °C EB > 500% TS~15–20 MPa WVP~3 g·mm/m^2^·d·kPa OP~60 cm^2^·mm/m^2^·d·atm	High flexibility Good biodegradability Thermal stability Good processing stability	Low transparency High production costs	Blowing film application Mulch film Cutlery Bags	[2,3,46]
TPS	EB < 100% TS < 5 MPa WVP~7–11 10^−10^·g/s·m·Pa Water solubility > 20%	Abundant renewable resources Biodegradable Cheap biopolymer	Poor thermal processability Low water vapor barrier Moisture sensitivity Retrogradation processes	Compost bags Food packaging Edible film Coating	[6,52,53,63]
PBS	EB~100–200%. TS~25 MPa. WVP~1.5 g·mm/m^2^·d·kPa OP~30 cm^2^·mm/m^2^·d·atm	High flexibility Excellent thermal stability	High stiffness High melt viscosity for processing Low transparency	Film (polymer blends) Film coating	[2,57,63]
PLA	T_m_~130–210 °C EB < 15% TS~20–60 MPa WVP~3 g·mm/m^2^·d·kPa OP~60 cm^2^·mm/m^2^·d·atm	High strength High transparency High processability	High brittleness Low heat distortion temperature Slow crystallization rate	Tray Bag Metallized and shrink films	[3,57,58]

T_m_ is melting temperature, EB is elongation at break, TS is tensile strength, WVP is water vapor permeability, and OP is oxygen permeability.

However, polymer blends have limits due to the incompatibility between polymers, giving poor mechanical and barrier properties. The properties of polymer blends depend strongly on the miscibility (or immiscibility) of the polymers. Compatibility is necessary to enhance the interfacial adhesion of the polymer blend, leading to a homogeneous structure with strong mechanical properties and high barrier and thermal resistance properties [3,64]. A compatibilizer is a compound used to reduce the interfacial adhesion energy and improve the adhesion between polymers. These compatibilizers have different responsibilities and functionalities, such as crosslink agent, plasticizer, and hydrolytic agent, leading to strong mechanical properties, a smooth surface structure, high water vapor and oxygen barriers, and high thermal stability [65]. Commonly used compatibilizers for TPS/polyester films are maleic anhydride, citric acid, itaconic acid, tartaric acid, and organic acid [66,67,68]. The compatibilizers used in PLA/PBS films include diphenyl diisocyanate, lysine triisocyanate, lysine diisocyanate, glycidyl methacrylate, benzoyl peroxide, organoclays, and epoxy functionality [57]. Compatibilizers can be extracted using essential oils such as carvacrol, citral, and α-terpineol to enhance the compatibility of PBAT/PLA and PBAT/PBS packaging [69,70].

## 4. Bio-Functional Packaging

Packaging plays an important role in the food supply chain. Four primary functions of packaging have been identified: (i) containment to move or transport something from one place to another place; (ii) protection from physical and chemical damage such as water, gases, microorganisms, dust, shocks, and force; (iii) convenience in responding to consumer demand and promoting products; and (iv) communication as a silent salesman to convey information to consumers [21].

Food packaging is used to protect food products from physical and chemical changes including heat, light, oxygen, moisture, pressure, and microorganisms while also preventing biological activity. Packaging prevents the spoilage and contamination of food products during storage, transport, and distribution [71]. Food marketing requires that the quality of the food products must be preserved, and food packaging plays a key role in reducing the impact of external factors and avoiding or delaying the deterioration of food quality. The role of food packaging, along with marketing needs, has led to the development of active or functional packaging that maintains or improves the quality of food products.

Active packaging is defined in European Regulation (EC) No. 450/2009 as “active materials mean materials and articles that are intended to extend the shelf life or to maintain or improve the condition of packaged food; they are designed to deliberately incorporate components that would release or absorb substances into or from the packaged food or the environment surrounding the food” [72]. Active packaging can be classified into release and scavenger/absorber systems, including antimicrobial, antioxidant-, or carbon-dioxide-releasing systems and systems absorbing oxygen, moisture, or ethylene, which prolong the shelf life or enhance the quality and safety of products [73]. Functional packaging is enhanced in its ability to maintain, improve, or modify the quality and safety of food products and also has modified or improved properties itself [71,74]. Functional packaging can be produced using functional compounds including natural extracts such as essential oil, herbs, and spices and synthetic chemical compounds such as potassium sorbate, citric acid, and nano-oxide compounds. Functional compounds are widely used to preserve food quality and agricultural products. Some properties of polymeric bioplastic packaging are listed in Table 3.

## 5. Functional Packaging for Meat Products

### 5.1. Antioxidant Packaging

Antioxidant packaging uses antioxidants to delay oxidation or maintain oxidation stability, leading to a reduction in quality deterioration in flavor and odor, as well as color and texture deterioration. Food additive antioxidants such as butylated hydroxytoluene (BHT) or butylated hydroxyanisole (BHA) incorporated into polyolefin film [84,85] may influence health hazards for consumers. An alternative approach now being widely studied is the use of natural extracts, synthetic chemicals, and nanoparticles as antioxidants. Many studies have demonstrated that antioxidant agents incorporated into bioplastic packaging effectively extend the shelf life of meat.

Chollakup et al. [86] studied the incorporation of rambutan peel extract and cinnamon oil as antioxidants into cassava starch and whey protein isolate films to preserve the quality of salami. The release of polyphenols from the rambutan peel extract and cinnamon oil gave strong antioxidant functions to the films, as confirmed using DPPH assay. Moreno et al. [87] investigated potato starch films containing the bioactive proteins lactoferrin and lysozyme, which acted as antioxidants. These films effectively reduced the lard oxidation of minced pork after long storage times (14 days) due to the strong chelation capacity of the transition metals in the bioactives, which inhibited the oxidation reaction, used as natural antioxidant preservatives. Fiore et al. [88] showed that rosemary essential oil incorporated into a chitosan–caseinate coating with a polylactic acid film prolonged the shelf life of fresh minced chicken, and 2% rosemary essential oil demonstrated the greatest radical scavenging activity. Panrong et al. [89] demonstrated that a bioplastic film containing green tea extract acted as an antioxidant. Green tea is a rich source of polyphenols, especially catechin, epicatechin, and epigallocatechin, which accelerated the formation of oxymyoglobin (redness) with its free radical scavenging capacity and reduced the lipid oxidation of bacon after storage for 20 days. Ribeiro Sanches et al. [90] examined the influence of the concentration of red cabbage extract and sweet whey as antioxidants due to their significant contents of phenolic compounds and anthocyanins. The results showed greater stabilization of oxymyoglobin after storing ground beef for 4 days. The interaction of the antioxidants from natural extraction was mainly due to their content of polyphenols, which can reduce oxidation and delay the quality deterioration of meat. Packaging can also incorporate sodium nitrite [74], ferulic acid [91], ethylenediaminetetraacetic acid [29], zinc oxide (ZnO) nanoparticles [92,93], pyrogallol [94], and gallic acid [95].

### 5.2. Antimicrobial Packaging

The growth of microorganisms influences product quality, shelf life, and safety. Thus, technology has been developed to maintain food quality and extend shelf life through the addition of antimicrobial agents that reduce or inhibit microorganism growth [11]. The basic mechanism involves the penetration of active compounds into the lipid structure of bacterial walls, leading to protein denaturation, cell membrane destruction, bacterial membrane leakage, and ultimately cell lysis. Furthermore, the efficiency of antimicrobial agents is highly dependent on their release ability, according to factors such as the concentration gradient and solubility with the food product [69].

Antimicrobial agents such as organic acids, essential oils, enzymes, plant extracts, and nanoparticles can be incorporated into bioplastic polymers to increase food packaging’s functional properties. Various types of antimicrobial packaging for extending the shelf life of meat products are presented in Table 4.

**Table 4:** Antimicrobial packaging produced using bioplastic polymers to extend the shelf life of meat products.

Packaging Material	Antimicrobial Agent	Product	Findings	Reference
PLA/PBAT	Cinnamaldehyde and tea polyphenols	Meat analogue	Films protective against *E. coli* and *S. aureus* during 10 days of storage.	[96]
PBAT/TPS	Nisin and ethylenediaminetetraacetic acid	Pork	Ethylenediaminetetraacetic acid and nisin protective against *L. monocytogenes*, *C. perfringens*, *S. aureus,* and *L. innocua.*	[29]
TPS/whey protein isolate	Rambutan peel extract and cinnamon oil	Salami	Rambutan peel extract and cinnamon oil displayed antibacterial activity against *B. cereus*, *E. coli*, and *S. aureus.*	[86]
PLA	Carvacrol	Ground beef	Carvacrol-loaded PLA film reduced TVC and extended shelf life at 1.1 ± 1.5 days.	[97]
PLA	Lauric arginate ester, sodium lactate, and sorbic acid	Meat	- Lauric arginate ester reduced *L. innocua*, *L. monocytogenes*, and *S. typhimurium* growth for 3–5 weeks - Sorbic acid reduced the growth of *L. innocua* but not *Salmonella*.	[98]
TPS	Gallic acid, chitosan, and carvacrol	Ham	- Chitosan and carvacrol acted synergistically in *L. monocytogenes* inhibition.	[99]
TPS/PBAT	Sodium nitrite	Pork	- Nitrite reduced TVC up to 1.5 log CFU/g. - Nitrite against Pseudomonas aeruginosa.	[74]
Chitosan	Thyme essential oil	Meat	- Thyme essential oil reduced yeast populations but did not affect aerobic mesophilic bacteria, lactic acid bacteria, and enterobacteria.	[100]
Chitosan	Aloe emodin	Pork	- Chitosan is a natural antibacterial agent with positively charged amino groups that interacted with microbial cell membranes. - Aloe emodin inhibited bacterial formation, bringing about bacterial metabolism disorder and death and protecting against *E. coli* and *S. aureus* as shown in Figure 7.	[101]
Sodium caseinate	Pomegranate peel extract	Ground beef	- Pomegranate peel extract protecting against *S. aureus* and *E. coli* enhanced the germicidal activity of sodium caseinate.	[102]
Carboxymethyl cellulose (CMC)	Encapsulated pomegranate extract	Fresh beef and chicken meat	- CMC film and encapsulated pomegranate extract interacted via hydrogen bonding at 3320 cm^−1^, which shifted to a lower wavenumber. - The film system was more effective in inhibiting *L. monocytogenes* than *C. jejuni*, *S. typhimurium*, and *S. aureus*. - The classical CMC could not retard the growth of bacteria until the end of storage, while CMC with encapsulated pomegranate extract could delay microbial growth in beef and chicken meat.	[103]
Cassava starch and sodium carboxymethyl cellulose	Caffeic acid and silica nanoparticles (C@SNPs)	Fresh beef and chicken meat	- Reduction in hydrogen bonds and hydroxyl groups of the film formed by the nanoparticles, reduced the hydrophilicity of the film matrix. - Film containing C@SNPs 5:1 could inhibit *E. coli* and *S. aureus* with percent reductions up to 65.43% and 61.90%, respectively. - The active packaging delayed the microbial growth and TBARS in the fresh meat. The TBARS in the meat packed in film were 0.130 mg MDA/kg, while in the control group, they were 0.611 mg MDA/kg.	[104]
PBAT/TPS	Zinc oxide (ZnO) nanoparticles	Pork	- ZnO delayed microbial growth and extended shelf life to more than 12 days. - ZnO against *S. aureus*, *E. coli*, Enterobacteriaceae, and *Pseudomonas* spp.	[105]
Bacterial nanocellulose (BNC)	Postbiotics of *Lactobacillus sakei*	Buffalo patty	- A slight shift in the stretching vibration of the carboxylic groups of the BNC (1627 cm^−1^) indicates the successful incorporation of postbiotics into the BNC matrix. - The growth of *L. monocytogenes* in the buffalo patty was significantly reduced by the BNC film containing postbiotics of *Lactobacillus sakei.*	[106]

Antimicrobial agents inhibit the growth of microorganisms, namely Gram-positive bacteria, Gram-negative bacteria, and fungi, through their incorporation with bioplastic matrices using coating, extruding, and casting. Novel functional packaging technologies can control and reduce microbial spoilage to better preserve the quality of and prolong the shelf life of meat.

### 5.3. Other Functional Packaging

**Figure 8:**
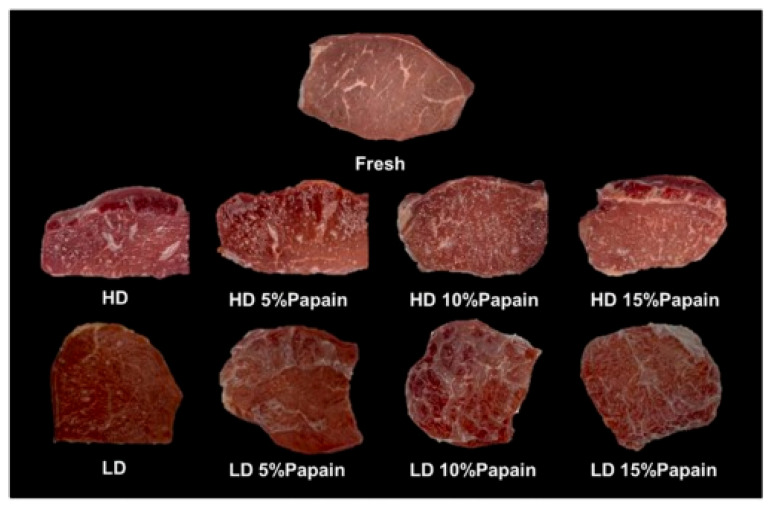
Appearance of packaged beef in starch films containing different papain concentrations (5%, 10%, and 15%) after incubation for 30 min. (Reproduced with permission from Wongphan, Khowthong, Supatrawiporn, and Harnkarnsujarit [107]).

**Figure 9:**
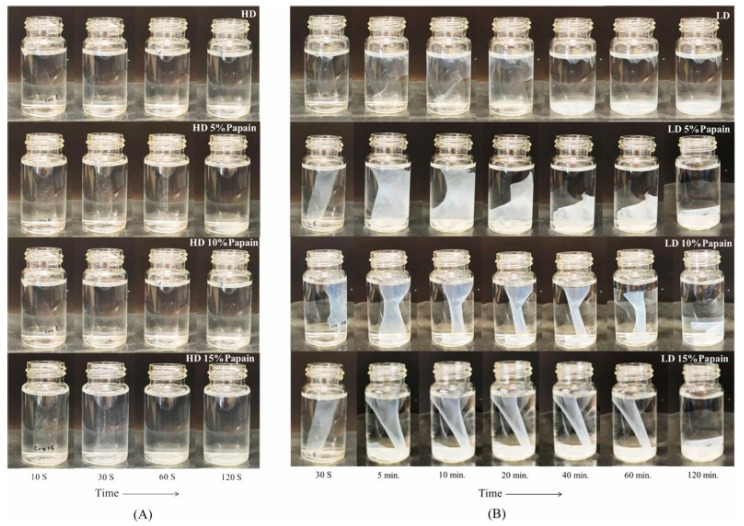
Water solubility as a function of time in starch films containing different papain concentrations (5%, 10%, and 15%). (Reproduced with permission from Wongphan, Khowthong, Supatrawiporn, and Harnkarnsujarit [107]). (**A**) high dissolution (HD)(**B**) low dissolution (LD).

Furthermore, functional packaging can also enhance other aspects of quality, such as tenderness, texture, and color. Wongphan et al. [107] developed a novel edible film made of starch incorporated with papain that improved the tenderness of beef, with reduced Warner–Bratzler shear values and hardness. Papain has proteolytic activities that hydrolyze collagen into myofibrillar proteins, leading to the degradation of the myofibrillar proteins. Enzymatic catalysis (papain) influenced the transformation and chemistry of myoglobin and thereby gave a bright red color. Moreover, the papain interacted with the starch film matrices, delaying the water dissolution of the starch films. The interaction between the starch and papain via hydrogen bonding reduced the binding between the starch and water, resulting in reduced dissolution, as shown in Figure 8 and Figure 9.

Chatkitanan and Harnkarnsujarit [28] reported that the incorporation of 1% and 2% sodium nitrite into LLPDE/TPS retained the hardness of the pork because the nitrite prevented protein aggregation and retained the protein structure. Therefore, incorporating active compounds into packaging improves the tenderness, maintains the protein structure, and extends the shelf life of meat.

## 6. Food Preservatives as Alternative Functional Compounds

Food preservatives are added to prevent food spoilage due to microorganisms (bacteria, mold, yeast, and fungi) and slow or prevent discoloration, flavor, or texture changes by delaying oxidation to maintain product freshness [108]. The food preservatives on product labels include ascorbic acid, butylated hydroxyanisole (BHA), butylated hydroxytoluene (BHT), calcium propionate, calcium sorbate, citric acid, ethylenediaminetetraacetic acid (EDTA), potassium sorbate, sodium benzoate, sodium erythorbate, sodium nitrite, and tocopherols (Vitamin E). These can be classified into chemical or synthetic preservatives and natural preservatives [109]. Both types of preservatives are commonly used in meat products, acting as antimicrobials and antioxidants. Natural preservatives are plant-based bioactive phenolic compounds found in plants, fruits, herbs, and spices. Essential oils or plant extracts, including rosemary, sage, thyme, oregano, cinnamon, clove extracts, green tea, and eugenol [110,111,112,113], are natural bioactive compounds that do not require a labeled additive (e-number) for their application in food products. Conversely, the amounts of chemical or synthetic preservatives used are limited by safety and toxicological requirements and regulated by legislation [108,114]. The functionality of some organic acids and their salts, commonly used in industry, is shown in Table 5, acting as antioxidant, antimicrobial, emulsifying, and stabilizing agents to maintain the freshness of appearance and consistency of products [115,116].

However, the direct addition of food preservatives to products impacts consumer health due to the residuals in the products, with the amount of residual food preservative being a significant factor to consider. Nevertheless, studies have shown that incorporating additives into packaging reduces the amount of additives remaining in the food compared to with their direct addition to food [28,74,105]. This serves as another option for maintaining food quality and enhancing consumer safety. Additionally, it helps address packaging-related issues, as shown in Table 6. The selection of active compounds must adhere to the standards for food contact materials, generally recognized as safe (GRAS) designation, and the specific regulations set by each country. When adding active compounds added to active packaging, their concentrations must align with the acceptable daily intake to ensure food safety. However, this should not compromise their ability to effectively preserve food products. Ideally, active compounds should be used at the minimum concentration that maximizes their efficiency while maintaining human safety and the desired packaging properties.

From these studies, it was found that research on incorporating food preservatives into food packaging through compression processes to enhance the efficiency of food packaging and increase consumer satisfaction and the development of packaging properties incorporating food preservatives is a new method for maintaining the quality and safety of meat products. This can be considered a new challenge emerging in the food and packaging industries. The current research on bioplastic meat packaging is primarily centered on scaling up production to the industrial level using extrusion technology. However, there is a need to more deeply investigate the stability of active packaging for meat products under various storage conditions (i.e., temperature, pressure, vibration) for specific durations of time. The storage conditions and duration can be directly linked to the meat supply chain (production, distribution, retail, and consumer behavior). Therefore, a comprehensive approach involving collaboration between marketing, supply chain stakeholders, and packaging developers is essential to address these challenges effectively.

## 7. Flavor/Odor Contamination

Contamination or migration are major issues in food packaging. Contamination or migration is defined as the transfer of compounds from packaging to food, which can affect its quality and safety in accordance with legislation or regulations. Contamination can alter the sensory and quality properties of food or cause harm to consumers’ health [126].

Flavor is an important sensory aspect of the overall acceptability of meat products [127]. The flavor of meat is attributed to a complex mixture of both volatile and non-volatile compounds. Organic natural volatile components include pyrazines, aldehydes, acids, ketones, hydrocarbons, esters, alcohols, nitrogen, and sulfur-containing compounds that are formed due to lipid oxidation and bacterial metabolism, leading to odorous sensations [128,129]. Non-volatile compounds produce a sense of taste by interacting with the taste buds on the surface of the tongue and in the mucous membranes of the palate and throat area [129]. Flavor can determine the quality of meat and poultry products. The volatile components derived from the degradation of proteins and lipids impact the sensory quality of meat depending on the degree of meat degradation [128].

Table 7 lists the volatile compounds in meat that impart specific product flavors or odors. The product packaging and conditions between storage and distribution also influence the formation of volatile compounds [130]. Some aromatic hydrocarbons in meat are derived from packaging due to migration. Song, Canellas, and Nerin [128] analyzed the volatile compounds in minced pork during storage in an active film (rosemary essential oil coated PET sheet) and identified 41 compounds using HS-SPME–GC–MS. These included alcohols, aldehydes, ketones, and hydrocarbons. Aromatic hydrocarbons such as toluene, ethylbenzene, m-xylene, o-xylene, isopropylbenzene, and p-cymene came from the packaging, indicating that the aromatic hydrocarbons in food contact material can be transferred into food and consequently pose a risk to consumers. Rivas-Canedo et al. [131] investigated how high-pressure processing (HPP) significantly changed the levels of some volatile compounds. The amount of alcohols and aldehydes decreased while 2,3-butanedione and 2-butanone were more abundant in the high-pressure-processed meats, with the migration of compounds from the plastic material such as branched-chain alkanes and benzene. Ref. [132] studied the volatile profile of Spanish salchichón when subjected to HPP using a multilayer plastic as the packaging material. The results showed high levels of compounds emanating from the plastic material, especially branched-chain alkanes and benzene. Wrona et al. [133] demonstrated that the incorporation of green tea extract into polyethylene using extrusion technology extended the shelf life of fresh meat, with epigallocatechin gallate, gallocatechin gallate, epicatechin gallate, gallocatechin, epigallocatechin gallate, and catechin gallate migrating from the packaging. Therefore, using active packaging as food contact materials should be studied in relation to the flavor (mass) transfer from packaging into products. The flavor of both volatile and non-volatile compounds affects the senses and can also impact food quality and product safety.

## 8. The Prospective Future of Functional Packaging Technology for Meat Products

Functional packaging technology for meat products is continuously evolving to address various challenges in the industry, including extending shelf life, preserving freshness, enhancing food safety, and meeting consumer demand for convenience.

**Shelf life extension**: One of the main challenges in meat packaging is extending shelf life while maintaining product quality and safety. Emerging technologies such as active packaging with natural compounds, extracted or synthesis compounds, and food preservatives such as antioxidant and antimicrobial agents can help extend shelf life.

**Sustainability**: With increasing environmental concerns and the aim of reducing food loss and waste, the demand for sustainable packaging solutions in the meat industry is therefore increasing. Future directions may include the development of biodegradable and compostable packaging materials derived from renewable sources, such as bioplastics.

**Active packaging**: Active packaging systems that include active ingredients such as antimicrobials, antioxidants, and oxygen scavengers directly added to the packaging materials are gaining attention. Future advances may involve the use of nanotechnology to increase the efficacy of the active ingredients and improve the kinetics of their release.

**Convenience and consumer interaction**: Packaging technology is also evolving to meet consumer demands for convenience and an enhanced user experience.

**Food safety and quality assurance**: Ensuring food safety and quality remains a paramount concern in the meat industry. Advanced packaging technologies such as barrier films, active coatings, and nanocomposites can help prevent contamination and maintain product freshness during storage and transportation.

**Regulatory compliance**: As the regulations governing food packaging become more stringent, manufacturers need to ensure that safety and labeling requirements are followed. Future directions may involve developing packaging materials that are compliant with emerging regulatory standards and guidelines.

## 9. Conclusions and Challenges

This study reviewed the recent advances in functional bioplastic meat packaging, focusing on enhancing the ability to maintain, improve, or modify the quality and safety of meat products. Meat products are crucial sources of protein and essential nutrients and play a significant role in supporting human health. However, their perishable nature poses challenges in terms of maintaining their quality and safety. Maintaining the optimal conditions during their production, storage, and distribution is essential for preserving the quality and safety of meat products to meet consumer expectations and regulatory standards. Advanced packaging technologies, proper handling practices, and stringent quality control measures play vital roles in mitigating spoilage and ensuring food safety throughout the meat supply chain. Further research is required to scale up polymeric-based packaging to industrial market production and promote a circular economy.

## Figures and Tables

**Figure 7:**
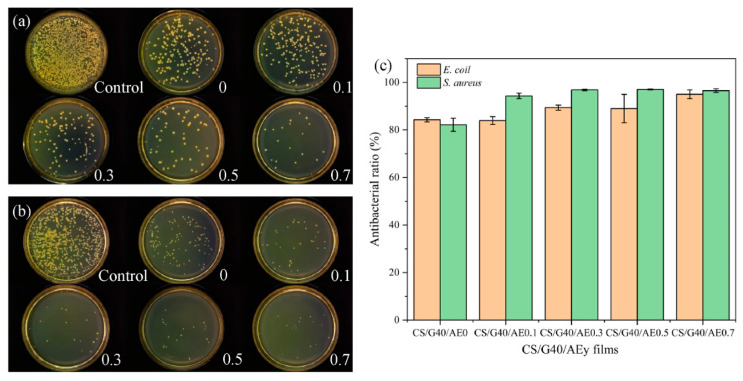
Antibacterial activity of chitosan, glycerol, and aloe emodin films against *E. coli* (**a**) and *S. aureus* (**b**); antibacterial ratio of chitosan, glycerol, and aloe emodin films (**c**). (Reproduced with permission from Yang, Ning, Ren, Xu, Li, and Wang [101]).

**Table 1:** Active packaging used to maintain or improve the color stability of meat.

Material	Additive or Condition	Food Product	Quality	References
Polypropylene trays	Modified atmosphere packaging: (i) HiOx MAP (80% O_2_/20% CO_2_) and (ii) CO MAP (0.4%CO/30%CO_2_/69.6% N_2_)	Beef steaks	Both MAP steaks developed bioprotective strategies to improve their color stability	[25]
Styrofoam trays	Modified atmosphere (70%O_2_ + 20%CO_2_ + 10% N_2_) and ascorbic acid, taurine, carnosine, and rosemary powder	Beef patties	- Rosemary powder and rosemary–ascorbic acid were effective in inhibiting the oxidation of both lipids and myoglobin - Ascorbic acid, taurine + ascorbic acid, and carnosine + ascorbic acid showed a small inhibitory effect on myoglobin oxidation	[26]
Multilayer polyolefin bag	Rosemary extract and sodium lactate with vacuum packaging	Ground ostrich meat	Rosemary extract retarded the formation of TBARS and showed the highest protection against discoloration	[27]
TPS/linear low-density polyethylene (LLDPE)	Incorporated sodium nitrite into film	Pork	Films containing nitrite effectively improved redness	[28]
PBAT/TPS	Incorporated nisin and nisin-ethylenediaminetetraacetic acid into film	Pork	PBAT/TPS films containing EDTA and nisin effectively inhibited lipid degradation, stabilized redness, and delayed meat discoloration	[29]

**Table 3:** Related research on the development of functional bioplastic films for food products.

Bioplastic Material	Functional Compound	Food Product	Packaging Properties	Food Quality	References
Corn starch/chitosan	Potassium sorbate and grapefruit seed extract	Bread	- Grapefruit seed extract films have high crystallinity, low hydrophilicity, low water vapor permeability, and high mechanical properties. - Potassium sorbate films showed high mechanical properties, high water vapor permeability than grapefruit seed extract films.	- Grapefruit seed extract showed the maximum zone of inhibition against *A. niger*. - Grapefruit seed extract extended the shelf life of bread after storage for 6 days.	[75]
PLA	Na^+^-montmorillonite	Salami	- Montmorillonite enhanced its water barrier properties.	- Montmorillonite reduced the lipid oxidation of processed meat products.	[76]
Whey protein	Ginger and rosemary essential oils	Minced lamb meat	- Increased essential oil content gave statistically high % of elongation and slightly decreased tensile strength.	- Incorporation of 1% essential oil significantly delayed microbiological deterioration of minced lamb meat.	[77]
PBAT/PLA	Carvacrol, citral, and α-terpineol essential oils	Pacific white shrimp	- Microstructures and water vapor and oxygen barrier properties were modified depending on the types and concentrations of the essential oils. - Citral conferred smoothness due to plasticization effects and improved compatibility.	- Citral and carvacrol effectively stabilized protein conformation in muscle tissues, delayed drip loss, and retained the adhesion between the shrimp cephalothorax and abdomen. - Essential-oil-compounded films prevented melanosis.	[69]
PLA/chitosan	Polyethylene glycol methyl ether methacrylate, stearyl methacrylate, and deoxycholic acid	Bread	- Functional compounds improved the compatibility of chitosan with PLA. - Deoxycholic acid was used as an antioxidant and antibacterial additive in PLA bioplastics.	- Deoxycholic acid was most efficient in inhibiting the growth of natural microorganisms on the bread slices stored in the packaging.	[78]
PBAT/TPS	Coconut nanocellulose, annatto, and citric acid	Freshly cut mango	- Nanocrystals reduced the water vapor permeability and influenced the increased stiffness of the films. - Tensile strength was dependent on the percentage of nanocrystals.	- The film maintained the characteristics of packaged Tommy Atkins mango for 14 days.	[79]
Gliadins (protein)	Cinnamaldehyde and natamycin	Cheese slices	- Incorporation of natamycin gave rise to films with a greater water uptake, weight loss, and diameter gain and higher water vapor and oxygen permeabilities. - Cinnamaldehyde enhanced the barrier properties of gliadin films due to the formation of a cross-linked matrix that restricted chain mobility and produced a more compact structure.	- Both compounds acted synergistically to prevent the growth of *A. niger*. The combination of cinnamaldehyde and natamycin gave rise to antifungal bioplastic films against common food spoilage microorganisms both in vitro and in cheese slices.	[80]
Chitosan	Montmorillonites and rosemary and ginger essential oils	Fresh poultry meat	- Reinforcement with montmorillonites improved chitosan barrier properties.	- Essential oil reduced oxidative processes in poultry meat. - Reinforcement with nanoclays reduced lipid oxidation and microbiological contamination, but the incorporation of essential oils only improved the barrier to oxidation.	[81]
PBAT/TPS	Titanium dioxide (TiO_2_)	Banana	- TiO_2_ nanoparticles increased mechanical strength and reduced oxygen, carbon dioxide, and water vapor permeability. - Films containing TiO_2_ also showed efficient oxygen-scavenging activity that removed residual oxygen from the package headspace.	- Banana fruit packaged in films containing TiO_2_ recorded a slower darkening color change and an enhanced shelf life with an increasing TiO_2_ content.	[82]
PBAT/TPS	Sodium benzoate and potassium sorbate	Noodle	- Sodium benzoate and potassium sorbate gave more homogeneous microstructures.	- Sodium benzoate and potassium sorbate effectively delayed *A. niger* and *Rhizopus* spp. and reduced the total viable count, yeast, and mold in fresh noodles. - Sodium benzoate and potassium sorbate improved the polymer compatibility but reduced the tensile strength and elongation.	[83]
TPS/PBAT	Nisin and ethylenediaminetetraacetic acid (EDTA)	Pork	- Nisin and EDTA improved the compatibility and adhesion networks, giving homo-structures.	- Nisin and EDTA showed efficiency in inhibiting microbial growth, stabilized the color of the packaged pork. - EDTA efficiently stabilized the lipid structures in wrapped meat muscles and retained the quality of the meat.	[29]

**Table 5:** Food preservative functionalities commonly used in industry.

Preservative	Functionality
Ascorbic acid	Oxygen scavenger
Citric acid	Enzyme inhibitor/metal chelator
Sulfites	Enzyme inhibitor/oxygen scavenger
Tocopherols	Free radical scavenger
Acetic acid	Disrupts cell membrane function (bacteria, yeasts, some molds)
Benzoic acid	Disrupts cell membrane function/inhibits enzymes (molds, yeasts, some bacteria)
Natamycin	Binds sterol groups in fungal cell membranes (molds, yeasts)
Nisin	Disrupts cell membrane function (Gram-positive bacteria, lactic-acid-producing bacteria)
Nitrates, nitrites	Inhibits enzymes/disrupts cell membrane function (bacteria, primarily Clostridium botulinum)
Propionic acid	Disrupts cell membrane function (molds, some bacteria)
Sorbic acid	Disrupts cell membrane function/inhibits enzymes/inhibits bacterial spore germination (yeasts, molds, some bacteria)
Sulfites and sulfur dioxide	Inhibits enzymes/forms additional compounds (bacteria, yeasts, molds)
Phosphate	Maintain juiciness and shelf life, emulsifying and stabilizing

**Table 6:** Functional bioplastic packaging incorporating food preservatives.

Material	Preservative	Method of Preparation	Findings	Reference
Gelatin	Ascorbic acid	Solution casting	- Ascorbic acid acted as an antioxidant and increased DPPH radical scavenging activity and total phenolic content. - Ascorbic acid had hydrogen donation capabilities and could scavenge free radicals by transferring electrons. - Ascorbic acid improved the flexibility related to a complex and dense microstructure.	[117]
Pectin (papaya)	Ascorbic acid	Solution casting	- Ascorbic acid acted as an antioxidant and prolonged the shelf life of pears. - Ascorbic acid provided weak films (low mechanical properties) and increased the aw values of papaya films due to its hydrophilic nature.	[118]
Calcium alginate	Acetic and propionic acid	Solution casting	- Organic acid acted as an antimicrobial against total coliform, *S. aureus*, lactic acid bacteria, and mold and yeast viable counts. - Alginate and organic acids showed H bonding between free water molecules and hydroxyl groups. - Films had strong mechanical properties but were more stretchable than the control films.	[119]
PBAT/TPS	Nisin and EDTA	Blown film extrusion	- EDTA and nisin acted as antimicrobials and inhibited *Listeria*, *C. perfringens*, *S. aureus*, and *L. innocua.* - The interaction between PBAT/TPS, EDTA, and nisin was alkylation. - EDTA and nisin improved the compatibility, as also shown by a smoother microstructure.	[29]
TPS/PBAT	Sodium nitrite and sodium erythorbate	Blown film extrusion	- Sodium nitrite and sodium erythorbate modified starch granule and increased compatibility. - Sodium nitrite and sodium erythorbate preserved ham quality via delay antioxidation.	[12]
Carboxymethyl cellulose	Potassium sorbate	Solution casting	- Potassium sorbate acted as an antimicrobial and inhibited *A. parasiticus*, *A. parasiticus*, and *A. flavus*. - The incorporation of potassium sorbate into carboxymethyl cellulose films increased the WVP values via blockage of the polymer matrix pores.	[120]
Chitosan/whey protein	Ascorbic acid, benzoic acid, and potassium sorbate	Solution casting	- Organic acid acted as an antimicrobial and delayed *S.* Typhimurium, *E. coli*, and *C. jejuni* in fresh-cut turkey for 6 days.	[121]
Protein	Sorbic or benzoic acids	Solution casting	- Organic acid acted as an antimicrobial and inhibited the growth of *E. coli* O157:H7, *L. monocytogenes*, and *S. aureus*. - Sorbic acid penetrated into protein more easily than benzoic acid, forming hydrogen bonds with amide groups of proteins. - Sorbic and benzoic acid gave high solubility.	[122]
TPS/PBAT	Sodium nitrite	Blown film extrusion	- Sodium nitrite acted as an antimicrobial and reduced total viable count, lactic acid bacteria and yeast and molds. - Interaction between the pork and TPS/PBAT film released nitrite from the film matrices and interacted with the myoglobin in the pork to form nitrosyl myoglobin, increased redness. - Sodium nitrite modified the C=O bonding of PBAT and improved its compatibility with TPS networks.	[74]
Starch	Potassium sorbate	Solution casting	- Potassium sorbate acted as an antimicrobial and delayed yeast growth. - Potassium sorbate formed complexes that modified the solubility, diffusivity and partition coefficients.	[123]
PBAT/TPS	Sodium benzoate and potassium sorbate	Blown film extrusion	- Organic acid acted as an antimicrobial and delayed *A. niger* and *Rhizopus* sp. growth. - Sodium benzoate and potassium sorbate modified the starch network and compatibility between PBAT and TPS.	[83]
PBAT	Sodium benzoate	Solution casting	- Sodium benzoate acted as an antimicrobial against *B. subtilis* and *S. aureus.* - Sodium benzoate gave higher barrier properties against water and methanol vapor to the PBAT film.	[124]
TPS/PBAT	Sodium metabisulfite	Blown film extrusion	- Sodium metabisulfite prevented mold growth and darkening in packaged salami. - Sodium metabisulfite prevented recrystallization. - Sodium metabisulfite formed S-O bonds with starch molecules.	[125]

**Table 7:** Volatile compounds in beef, pork, and poultry and their odor data, modified from [128,134].

Compound Name	Characteristic Flavors/Aromas
Benzaldehyde	Volatile almond oil, bitter almond, burning aromatic taste
Benzene	Pleasant, distinct
sec-Butanamine	Seafood, green, onion
Butenal	Malty, green, roasted
n-Caproic acid	Goaty
3-Carene	Sweet and pungent odor but more agreeable than turpentine, orange peel, lemon, resin
Cyclobutanol	Roasted
2,2,6-Trimethylcyclohexanone	Mint, acetone
2,4-Decadiena	Deep fatty flavor, chicken flavor at 10 ppm, citrus/orange/grapefruit flavor at lower dilutions
Decanal	Powerful, waxy, aldehydic, orange, citrus peel
2-Decenal	Tallow, orange
1,3-Bis(1,1-dimethylethyl) benzene	Cooked beef
N,N 0-Dimethyl 1,2-ethanediamine	Ammonia
5-Ethylcyclopent-1-enecarboxaldehyde	Fragrant, perfume
2-Pentylfuran	Green bean, butter
2,4-Heptadienal	Nut, fat
Heptanal	Oily, fatty, rancid, unpleasant, penetrating fruity odor in liquid
1-Heptanol	Fragrant, woody, oily, green, fatty, winey, sap, herb
2-Heptanone	Fruity, spicy, cinnamon, penetrating fruity odor in liquid
6-Methyl 2-heptanone	Cloves, menthol, eugenol
2-Heptenal	Soapy, fatty, almond, fishy, unpleasant
Hexanal	Fatty, green, grassy, strong green, tallow, fat, unripe fruit when dilute
Hexane	Faint peculiar odor
Hexanol	Woody, cut grass, chemical/winey, fatty, fruity, weakly metallic, green
2-Ethyl 1-hexanol	Resin, flower, green
2-Hexen-1-ol	Green, sharp, leafy, fruity, unripe banana
3-Methylbutanal	Pungent apple-like odor, malt
Limonene	Pleasant, lemon-like, turpentine, citrus, fruity, fresh, light
Methyl salicylate	Cooling sensation, wintergreen, gaultheria
2,4-Nonadienal	Fat, wax, green, watermelon, geranium, pungent
Nonanal	Floral, citrus, fatty, grassy, waxy, green
2-Nonanone	Hot milk, soap, green, fruity, floral
2-Nonenal	Cardboardy, orris, fat, cucumber
Octadecanal	Oil
Octanal	Harsh, fatty, orange peel, soapy, lemon, green, honey
1-Octanol	Penetrating aromatic odor, fatty, waxy, citrus, oily, walnut, moss, chemical, metal, burnt
2-Methyl 3-octanone	Herb, butter, resin, gasoline
2-Octenal	Green, nut, fat
(Z)-3-Octene	Fruity, old apples
1-Octen-3-ol	Mushrooms, compound excreted by many insects
2-Octen-1-ol	Green citrus
3-Octen-2-one	Nuts, crushed bugs, earthy, spicy, herbal, sweet, mushroom, hay, blueberry
Pentanal	Almond, malt, pungent, acrid, fermented, fruity
Pentane	Very slight warm flavor, oxidized
1-Pentanol	Mild odor, fuel oil, sweet, fruity
5-Amino 1-pentanol	Mild
α-Pinene	Piney, fruity, citrus, turpentine
β-Pinene	Pine, citrus, fruity, resin, turpentine
Piperazine	Salty
Propanol	Alcoholic
Styrene	Penetrating odor, sweet smell
Tetradecane	Alkane
Tridecane	Alkane
2-Tridecenal	Sweet, strong, spicy
Ethanol	Sweet, alcoholic
2,3-butanedione	Butter, pungent
Acetic acid	Sour, vinegar
3-Hydroxy-2-butanone	Sweet, fatty
3-Methyl-1-butanol	Alcoholic, fruity
4-Methyl-2-pentanone	Green, herbal, fruity
Toluene	Paint
2,3-Butanediol	Buttery
Ethylbenzene	Paint
m-Xylene	Plastic
1-Hexanol	Sweet, alcoholic
5-Methyl-4-hepten-3-one	Fruity
Decane	Gasoline
p-Cymene	Citrus, woody
2-Ethyl-1-hexanol	Floral, sweet
Benzeneacetaldehyde	Floral, sweet
1-Octanol	Green, orange
Tridecane	Gasoline
Dodecane	Gasoline
(Z)-2-Octen-1-ol	Sweet

## Data Availability

The data presented in this study are available on request from the corresponding author (due to privacy).
